# MRI Findings of Extradural Fat in Patients with Cauda Equina Syndrome: A Novel Perspective

**DOI:** 10.1007/s43465-025-01406-5

**Published:** 2025-05-13

**Authors:** Xianping Luo, Deng Li, Yi Zhai, JingLi Qian, Caiyun Ying

**Affiliations:** 1 Department of Radiology, People’s Hospital of Chongqing Liangjiang New Area, No.199 Renxing Road, Renhe Street North New District, Yubei District, Chongqing, 401120 China; 2 Department of Traditional Chinese Medicine and Rehabilitation, People’s Hospital of Chongqing Liangjiang New Area, No.199 Renxing Road, Renhe Street North New District, Yubei District, Chongqing, 401120 China

**Keywords:** Spinal epidural lipomatosis(SEL), Redundant nerve roots (RNRs), Magnetic resonance imaging (MRI), Epidural fat, Correlation

## Abstract

**Objective:**

To explore the manifestations of extradural fat in patients with Redundant nerve roots (RNRs) via lumbar MRI.

**Methods:**

A total of 492 patients with RNRs were enrolled and divided into two groups: Group A (*n* = 459) with known potential causes of RNRs, such as lumbar spondylolisthesis, scoliosis, disc herniation, and ligamentum flavum hypertrophy; Group B (*n* = 33) without the aforementioned causes; and control Group C (*n* = 33) with age- and sex-matched RNRs-negative patients. MRI was used to assess the morphology of extradural fat and the appearance of the cauda equina and to measure the subcutaneous fat thickness in the lumbar-sacral region (LSFTT). Statistical analysis was performed via t tests, chi-square tests, and Wilcoxon signed-rank tests, with *p* < 0.05 considered statistically significant.

**Results:**

In Group A, there was no statistically significant difference in the LSFTT among the different grades of extradural fat. In Group B, the incidence rates of Grade 2 and Grade 3 extradural fat were 66.7% and 24.2%, respectively, which were significantly greater than the 27.3% and 0% reported in Group C, with a statistically significant difference between Groups B and C (*p* < 0.001). The correlation coefficient between increased extradural fat and RNRs was 0.669 (*p* < 0.001). There were no statistically significant differences in the morphology or relative length of the RNRs among the different grades of extradural fat in Group B.

**Conclusion:**

MRI is an important tool for assessing extradural fat and RNRs, providing crucial information for clinical decision-making and improving patients’ prognoses. Increased extradural fat may contribute to the development of RNRs and should be a concern for clinicians.

## Introduction

Spinal epidural lipomatosis (SEL) is caused by the excessive deposition of normal, unencapsulated fat tissue in the extradural space, leading to compression of the dural sac and spinal nerve roots. The global incidence rate ranges from approximately 2.5–16.7% [1]. It was first described in 1975 by Lee et al. [2] in patients treated with exogenous glucocorticoids after kidney transplantation. The mechanism of SEL is unclear and may be related to factors such as exogenous corticosteroid overuse [3], obesity [4], endogenous steroid overdose [5], nonalcoholic liver disease [6], and idiopathic disease. Therefore, metabolic syndrome, diabetes, obesity, and Cushing's disease have become risk factors related to the occurrence and development of this disease. With the global increase in overweight and obese patients, obesity is becoming a major risk factor. The early symptoms are often chronic low back pain, which can last for months or years. It is easily misdiagnosed as symptoms caused by other diseases, such as lumbar disc herniation, resulting in an underestimation of the disease. Patients in this period are often treated conservatively with weight loss, reduction or withdrawal of steroids [7], and a reduction in the amount of epidural fat to improve their symptoms. The later stage mainly manifests as radicular signs and symptoms related to spinal canal stenosis, such as low back pain, radicular pain of the lower limbs or neurogenic claudication [8]. As a result, the quality of life of patients is significantly reduced, medical expenses are increased, and even acute exacerbations occur in some cases [9, 10] requiring emergency surgical treatment. The commonly used surgical procedure is laminectomy or laminoplasty, which is performed by removing excessive fat deposited in the epidural capsule [11]. Valentina Tardivo et al. [12] found that the cauda equina nerve in an SEL patient with rapid deterioration of neurological symptoms after the failure of conservative treatment was edematous, thickened, and tortuous, which manifested as redundant nerve roots (RNRs). However, previous studies [13–15] have shown that patients with lumbar spinal stenosis combined with RNRs have worse preoperative and postoperative scores than those without RNRS, and the cure rate is lower. The RNRs are considered to be one of the reasons for the poor prognosis of patients. Therefore, early diagnosis and timely evaluation of the occurrence and development of SEL are of great clinical significance to help patients seek timely medical treatment and avoid the occurrence of serious adverse consequences.

At present, the diagnosis of SEL is mainly based on a history of characteristic nerve root compression, clinical symptoms, and imaging studies. Magnetic resonance imaging (MRI) technology has become a reliable imaging method for the diagnosis of lumbar spine diseases due to its good soft tissue resolution, multiparameter measurement, and multisequence and multiplane imaging methods, making it a key player in surgical decision-making.SEL shows continuous spindle-shaped fat deposition in the epidural capsule of the spinal canal on the sagittal MR plane, which is characterized by a high signal intensity on T1 WI and T2 WI and a low signal intensity on the T2 fat-suppression sequence [4]. Some scholars [16] have proposed using the absolute value of the anteroposterior diameter of the epidural fat and the ratio of the anteroposterior diameter to the anteroposterior diameter of the dural sac to analyze the morphology of the spinal canal. This measurement method is complicated and time-consuming. Ishikawa [17] proposed a grading based on the morphology of fat on MRI sagittal images, which can be divided into three grades:grade 1 was defined as epidural fat observed within the border between the anterosuperior edges of the upper and lower neighboring neural arches; grade 2 was defined as fat observed over the border at the middle but not at the edges of neural arches on both sides and grade 3 was defined as fat observed over the border at the edges of neural arches on at least one side. Although this grading method is nonquantitative, it can be used to intuitively and quickly determine whether the amount of epidural fat is increased and the extent of the increase.

Although there are many quantitative and nonquantitative methods for detecting intraspinal epidural fat, the imaging diagnostic criteria for SEL have not been unified, and no further research has been conducted on changes in the cauda equina nerve in the spinal canal. There are few studies on the correlation between epidural fat and the RNRs. Therefore, it is particularly important to determine whether RNRs can lead to serious consequences in order to achieve early and accurate diagnoses of SEL.

Based on the main factors affecting the surgical prognosis of patients—the overall function of the cauda equina nerve—the grading performance of extradural sac fat in lumbar MR images using the RNRs as the reference standard was analyzed in this study to achieve early and accurate diagnosis of SEL, help spine surgeons determine risk groups and provide strong imaging evidence for patients to choose appropriate treatment methods and improve the clinical cure rate.

## Materials and Methods

### Subjects

**Fig. 1:**
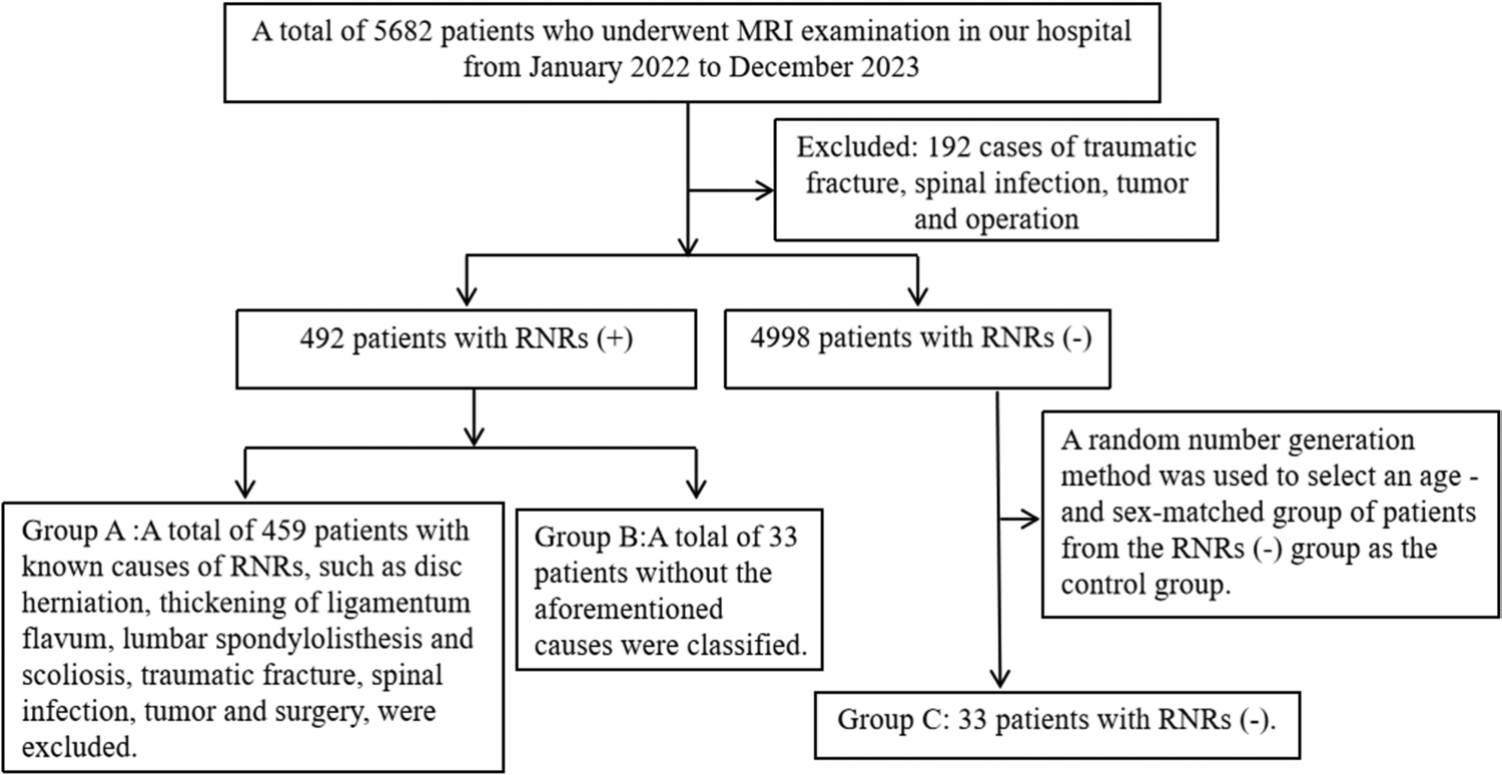
Inclusion and exclusion criteria

A retrospective collection of data from 5,682 patients who underwent lumbar spine MRI examinations at our hospital from January 1, 2022 to December 31, 2023 was conducted. Patients whose MR images met the inclusion and exclusion criteria were classified into the RNRs (+) group. They were grouped according to common causes, with patients having known potential causes of RNRs, such as lumbar spondylolisthesis, scoliosis, intervertebral disc herniation, and thickening of the ligamentum flavum classified as Group A, whereas patients without the aforementioned causes were classified as Group B. The patients classified as RNRs (−) served as a matched control group from which we selected an age- and sex-matched patient group as Group C via a random number generation method (Fig. 1).

The inclusion criteria were as follows: RNRs showing thickening, buckling, and serpentine- or loop-shaped structures on their T2 WI MR sagittal images.

The exclusion criteria were as follows: patients with known possible causes of RNRs, such as lumbar disc herniation, ligamentum flavum hypertrophy, spondylolisthesis, scoliosis, traumatic fractures, spinal infections, tumors, or surgery.

### MRI Imaging

A SIEMENS 1.5 T magnetic resonance imaging scanner was used, with a spinal matrix coil selected, and the subject was placed in a supine position with the lower limbs straightened and the head elevated, which is the conventional position for magnetic resonance imaging of the lumbar spine. The scanning range included the upper edge of the T12 vertebra to the level of the lower edge of the S2 vertebra.

### Measurement and Observation of Relevant Indicators

Measure the thickness of lumbar subcutaneous fat tissue (LSFTT, mm) on MRI images; assess the morphology of cauda equina redundancy and measure relative length; evaluate the grading of lumbar epidural fat on T1-weighted sagittal images.

#### The Thickness of Lumbar Subcutaneous Fat Tissue (LSFTT, mm)

**Fig. 2:**
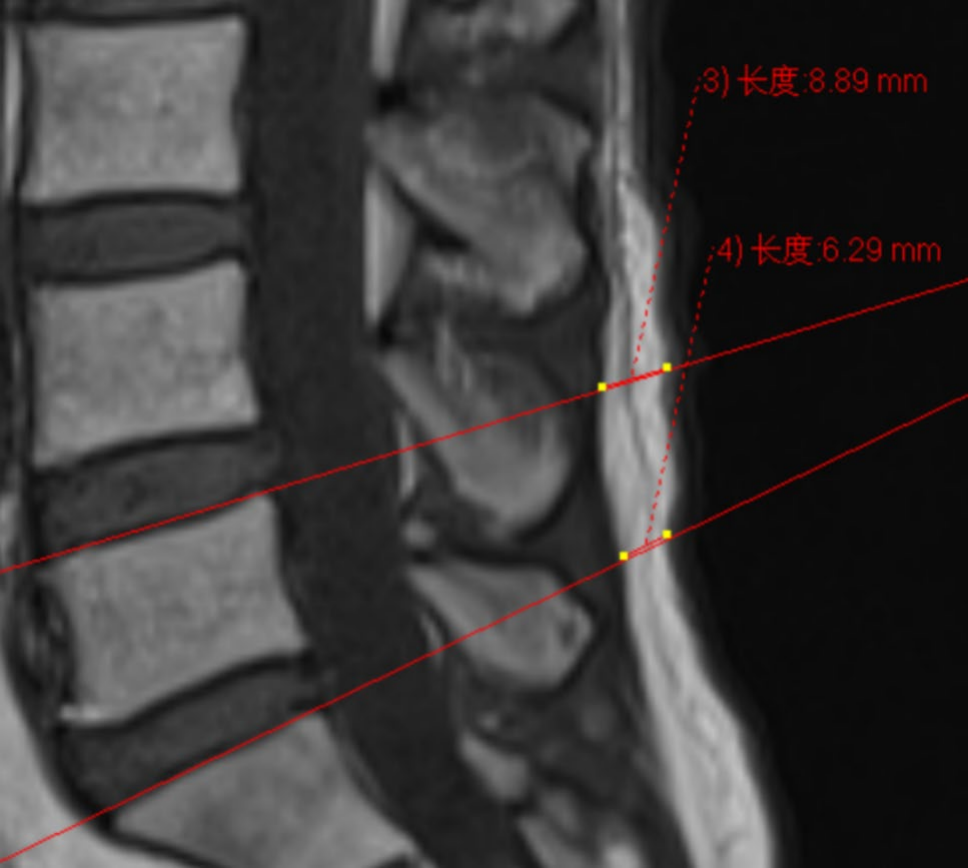
Mid-sagittal T1-weighted magnetic resonance image shows the measurement of lumbar subcutaneous fat tissue thickness in a Patient

LSFTT on MR images was measured, the morphology of cauda equina redundancy was assessed, the relative length was measured, and the grading of lumbar epidural fat on T1-weighted sagittal images was evaluated. As shown in Fig. 2.

#### Imaging Manifestations and Typing of RNRs

**Fig. 3:**
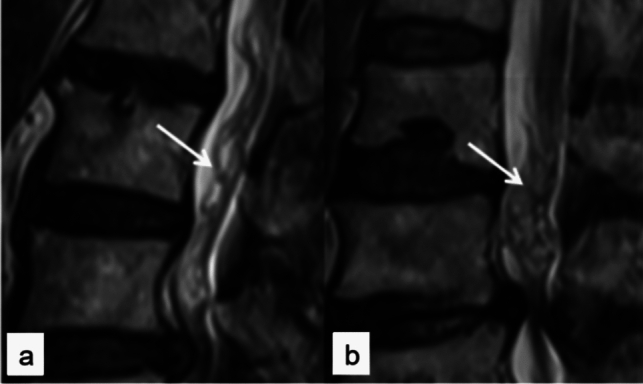
Variety of RNRs forms: loop shaped (**a**); serpentine shaped (**b**)

RNRs exhibit thickening, buckling, and serpentine- or loop-shaped structures on T2 WI MR sagittal images. Poureisa et al. [18] divided RNRs into loop-shaped and serpentine-shaped according to the morphological characteristics of the RNRs forms. As shown in Fig. 3.

#### Relative Length of the RNRs

**Fig. 4:**
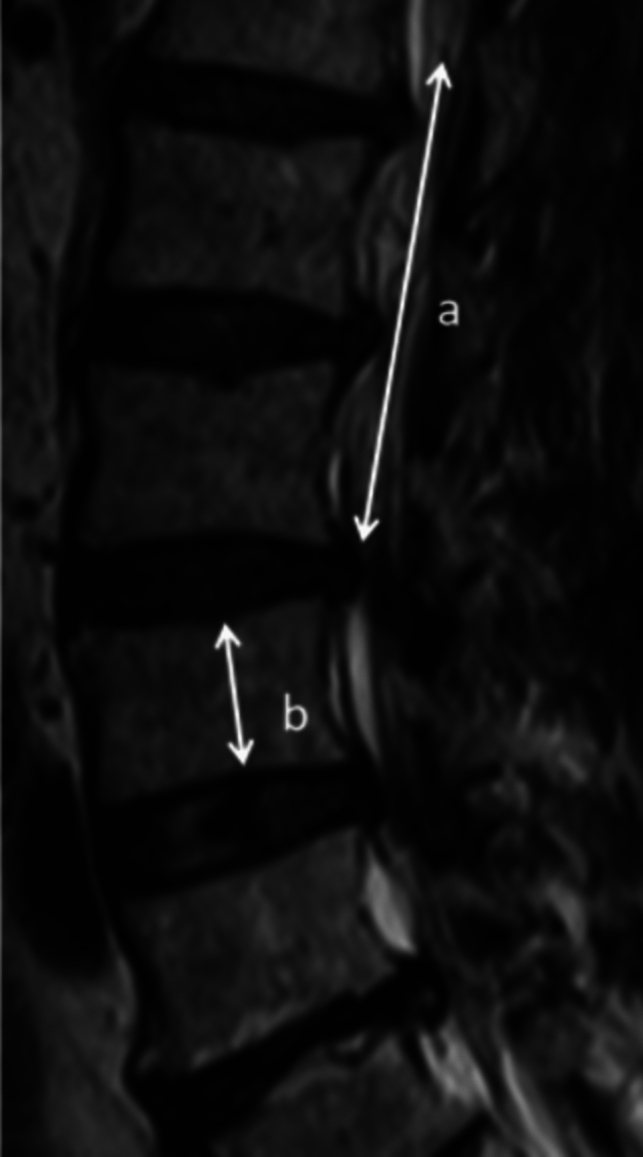
The median sagittal T2 WI of the lumbar spine shows the RNRs, where a is the length of the RNRs, b is the height of the fourth lumbar spine, and the relative length of the RNRs = $\frac{{\varvec{a}}}{{\varvec{b}}\boldsymbol{ }}$

The length of the RNRs was measured along the T2 WI mid-sagittal plane of the lumbar spine and is expressed as the relative length of the RNRs, where the relative length of the RNRs = the length of the RNRs/the height of the fourth lumbar spine. To ensure homogeneity in this study, only the height of the fourth lumbar spine was selected as the reference height. When the fourth lumbar spine was flattened or severely diseased, it was not sufficient to represent the height of the vertebral body, and the adjacent relatively normal vertebral body was selected for height measurement. As shown in Fig. 4.

#### Sagittal Grading of Epidural Fat

**Fig. 5:**
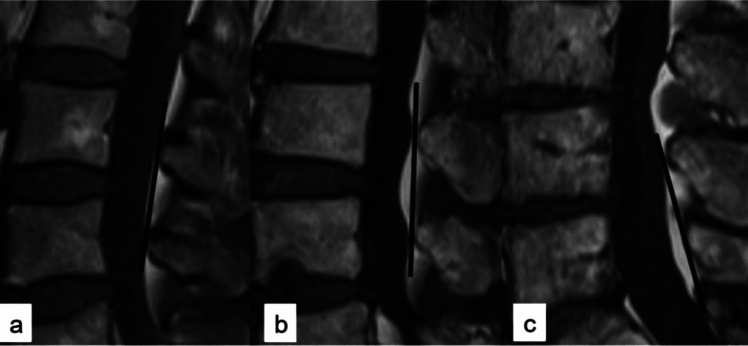
Sagittal grading of epidural fat on T1-weighted MR images: **a**:Grade 1 was defined as epidural fat observed within the border between the anterosuperior edges of the upper and lower neighboring neural arches. **b** Grade 2 was defined as fat observed over the border at the middle but not at the edges of neural arches on both sides. **c** Grade 3 was defined as fat observed over the border at the edges of neural arches on at least one

Ishikawa et al. [17] classified epidural fat on MRI sagittal images, which can be divided into three grades. Morphological characteristics of epidural fat recognisable on MR images from L1-2 to L5-S1 were assessed using the lumbar SEL grading system. These classifications and gradings represent the presence and severity of epidural fat compression of the dural sac as shown in Fig. 5.

**Fig. 6:**
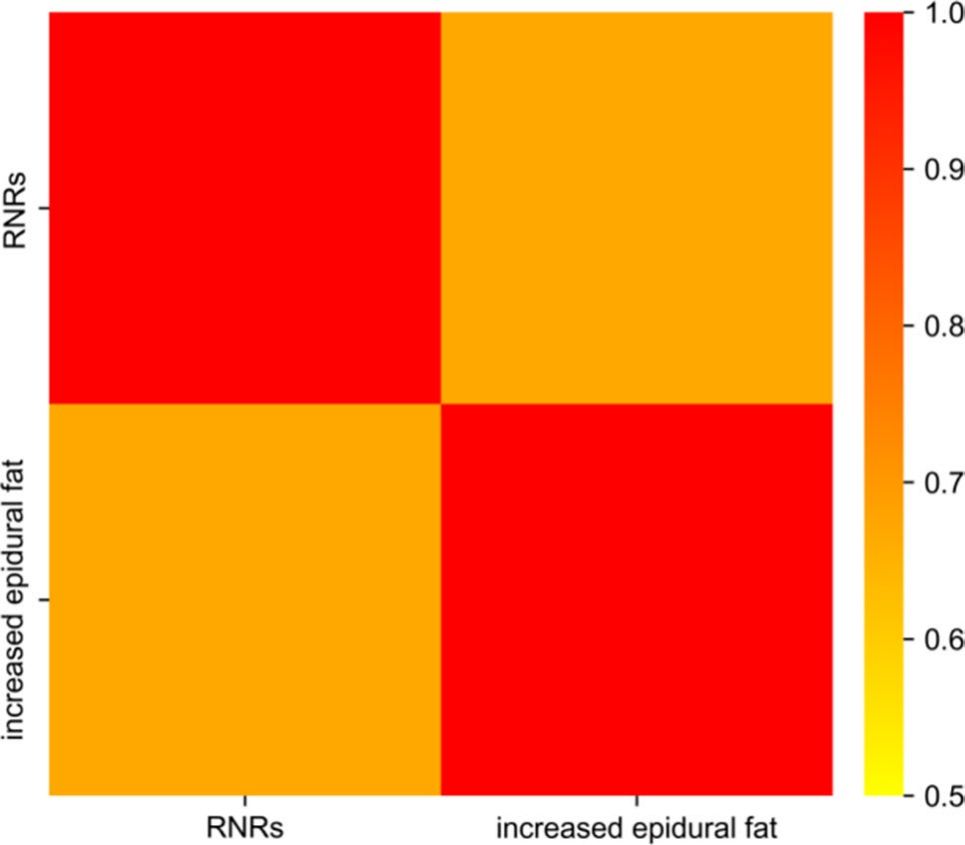
Correlation heatmap

**Fig. 7:**
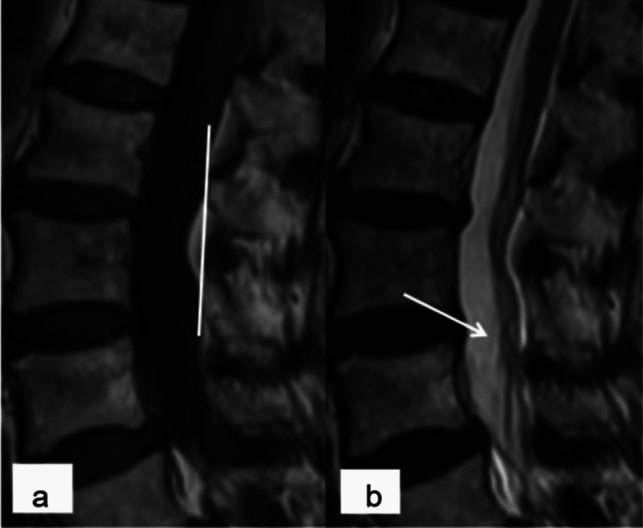
An 83-year-old female patient was admitted to the hospital due to recurrent low back pain and leg pain for 8 years and aggravation for 1 month. **a** Anterior fat appeared as grade II on T1 WI sagittal images, with a curved indentation at the posterior edge of the dural sac. **b** The RNRs showed thickening, buckling, and a serpentine or loop shape on T2 WI sagittal images

Before the experiment began, three physicians with over 15 years of experience in imaging diagnosis, including two associate chief physicians and one attending physician, established uniform regulations for the measurement methods. The measurement data were independently evaluated and measured by one attending physician and one associate chief physician, with the measured values averaged for statistical analysis. Another associate chief physician conducted a further review of the results, and in cases of inconsistency, the three physicians consulted to achieve consensus (Figs. 6 and 7).

### Statistical Analysis

The data were analyzed using SPSS Statistics 26.0. Continuous data are expressed as the mean ± standard deviation ($\overline{\text{x} }$± *s*) and were compared using a t test or analysis of variance; categorical data are expressed as the frequency (percentage) [*n* (%)], and unordered categorical variables were compared using the chi-square test or Fisher's exact test. The Wilcoxon signed-rank test was used to compare whether the variables were significantly different between the two groups after matching; *p* < 0.05 was used to indicate statistical significance.

## Results

Group A had a total of 459 cases (93.3%). Based on the grading of epidural fat in the sagittal plane, a one-way ANOVA was conducted, and we found no difference in the LSFTT among the different grading groups of epidural fat. See Table 1 for details.

**Table 1:** Correlation between epidural fat sagittal plane grading and LSFTT (*n* = 459)

Variant_*g*_	Total	Sagittal grading of epidural fat			*F*	*p*
		1	2	3		
	492 (100.0%)	192 (39.0%)	224 (45.5%)	76 (15.4%)		
L4/5 disc level:LSFTT (mm)	12.959$\pm$8.1171	12.613$\pm$7.5781	12.894$\pm$8.6494	14.025$\pm$7.8218	0.837	0.434
L5/S1 disc level:LSFTT (mm)	12.613$\pm$8.9875	14.064$\pm$8.2132	14.061$\pm$9.9322	14.757$\pm$7.9555	0.191	0.826

^*^p value ≤ 0.05 is statistically significant

LSFTT:The thickness of lumbar subcutaneous fat tissue

A total of 33 patients who met the inclusion and exclusion criteria, including 10 males (30.3%) and 23 females (69.7%), aged 29–89 years, with an average age of 62.88 years, were included in the RNRs (+) group. There was no significant difference in general information between the RNRs (+) group and the RNRs (−) group (*p* > 0.05). For details, see Table 2.

**Table 2:** Descriptive statistics between groups (*n* = 66)

Variant	Total ($\overline{x }$±s)/*n *(%)	RNRs^a^		*Z* value	*P* value
		Group C ($\overline{x }$±*s*)/*n* (%)	Group B ($\overline{x }$±*s*)/*n* (%)		
	66 (100.0%)	33 (50.0%)	33 (50.0%)		
Sex				0.000	1.000
Male	20 (30.3%)	10 (30.3%)	10 (30.3%)		
Female	46 (69.7%)	23 (69.7%)	23 (69.7%)		
Age	62.88 (55.75$,$73.25)	62.88 (55.50$,$73.50)	62.88 (55.50,73.50)	0.000	1.000
Sagittal grading of epidural fat				−4.284	< 0.001*
1	27 (40.9%)	24 (72.7%)	3 (9.1%)		
2	31 (47.0%)	9 (27.3%)	22 (66.7%)		
3	8 (12.1%)	0 (0.0%)	8 (24.2%)		

^*^p value ≤ 0.05 is statistically significant

^a^ RNRs: Redundant nerve roots

The relationship between the morphology of epidural fat and the occurrence of RNRs was analyzed by the Wilcoxon signed rank test. There was a statistically significant difference in the distribution of epidural fat between the RNRs(+) group and the RNRs(–) group (*p* < 0.001). The total percentage of patients with fat grades 2 and 3 in the RNRs(+) group was 90.9%, which was significantly greater than that in the RNRs(–) group. For details, see Table 2.

Sagittal grading of epidural fat in patients with RNRs: Patients were grouped according to fat grade in the RNRs (+) group. Sex and RNRs morphology were compared by a test (corrected by Fisher's exact test). Sex (*p* = 0.300) and RNRs morphology (*p* = 0.139) were not considered to be differentially distributed among the different fat grades. Age and redundancy relative length were tested by a non-parametric test (Kruskal‒Wallis *H* test) because the sample size of each group was small after grouping. There was no significant difference in age (*p* = 0.579) or relative length of redundancy (*p* = 0.407) between the different fat grades. For details, see Table 3.

**Table 3:** Intra-group differences in Group B

Variant	Total ($\overline{x }$± *s*)/*n* (%)	Sagittal grading of epidural fat			${\chi }^{2}$/*H* value	*P* value
		1 grade	2 grade	3 grade		
	33 (100.0%)	3 (9.1%)	22 (66.7%)	8 (24.2%)		
Sex					2.411	0.300
Male	10 (30.3%)	0 (0.0%)	6 (27.3%)	4 (50.0%)		
Famale	23 (69.7%)	3 (100.0%)	16 (72.7%)	4 (50.0%)		
Age	62.88 (55.50, 73.50)	57.00 (53.00, 61.00)	65.09 (56.00, 74.00)	59.00 (50.00, 69.00)	1.091	0.579
RNRs^a^ form					3.209	0.139
Loop shaped	30 (90.9%)	2 (66.7%)	21 (95.5%)	7 (87.5%)		
Serpentine shaped	3 (9.1%)	1 (33.3%)	1 (4.5%)	1 (12.5%)		
Relative length of RNRs	3.82 (2.835, 4.495)	4.49 (4.040, 4.985)	3.87 (2.780, 4.760)	3.42 (2.945, 4.055)	1.796	0.407

^*^p value ≤ 0.05 is statistically significant

^a^ RNRs: Redundant nerve roots

Spearman correlation was used to analyze the correlation between the RNRs and sagittal grade of the epidural fat. The correlation coefficient between increased epidural fat and the RNRs was 0.669 (*p* < 0.001). For details, see Table 4 and Fig. 5.

**Table 4:** Correlation analysis between RNRs and increased epidural fat

	RNRs	Increased epidural fat
RNRs	1.00	
Increased epidural fat	0.669^***^	1.00

^***^p < 0.001

RNRs:Redundant nerve roots

## Discussion

We conducted a retrospective analysis of 492 patients with RNRs, among which 459 cases (approximately 93.3%) included known literature-reported potential causes of RNRs, such as lumbar spondylolisthesis, scoliosis, intervertebral disc herniation, and thickening of the yellow ligament. Moreover, 33 patients (approximately 6.7%) had RNRs without the aforementioned causes. These patients often had mild clinical symptoms and did not show common causes on MRI, although SEL itself is a rare and often overlooked condition, leading to missed opportunities for early and timely treatment. As their condition progressed, they had to undergo surgical decompression, which undoubtedly increased their suffering. Therefore, we should pay more attention to RNRs patients, especially those without common causes of MRI, and consider the possibility of SEL. The innovation of this study lies in the new perspective on the relationship between epidural lipomatosis (SEL) observed through lumbar MRI and RNRs, providing valuable insights into the relationship between epidural fat and RNRs, which can help spinal surgeons identify high-risk patients and choose appropriate treatment strategies to improve clinical outcomes.

Increasing evidence suggests that changes in the volume of epidural fat in the spine vary with changes in Body Mass Index (BMI). Although BMI is a relatively simple measurement method, it still requires calculating the patient's weight and height, which may increase the limitations of retrospective studies. The purpose of this study involved finding a simpler method by conducting basic measurements (subcutaneous fat thickness in the lumbar and sacral regions) on lumbar MR images to explore the correlation with changes in epidural fat volume. We measured the subcutaneous fat thickness at the L4/5 and L5/S1 intervertebral disc levels in the Group A patients, but the results revealed no significant correlation between the deposition of lumbar epidural fat and subcutaneous fat deposition. Although the proportion of female patients in Group B (69.7%) was significantly greater than that of male patients, consistent with previous reports [19, 20], we found no difference in sex or age across different fat grades (*p* = 0.3, *p* = 0.579). The reasons for these results may be that the deposition of fat tissue in different locations is influenced by various factors, including genetics, sex, diet, lifestyle, and hormone levels. For example, women tend to accumulate more fat in the hips and thighs, whereas men are more likely to accumulate fat in the abdomen. Future studies may need to incorporate more factors to explore the reasons for the deposition of lumbar epidural fat.

Our results showed that the proportion of patients in Group B with epidural fat grades of 2 and 3 was significantly greater than that in Group C (*p* < 0.001). The correlation coefficient between increased epidural fat volume and RNRs was 0.669, which was significant (*p* < 0.001). These results suggest that increased epidural fat may contribute to the development of RNRs, emphasizing the importance of early and accurate diagnosis of SEL. Normal epidural fat appears as uneven but smooth and discontinuous distributions of fat signals, and as fat tissue continues to accumulate in the epidural space, it bulges toward the dural sac, compressing the posterior edge of the dural sac, causing varying degrees of indentation. When the cauda equina is subjected to long-term pressure from epidural fat tissue, repeated pulling and functional abnormalities of microvessels within the fat tissue lead to ischemia and hypoxia of the cauda equina, resulting in tortuous deformation and the occurrence of RNRs, which aligns with the mechanisms of RNRs formation [21]. Previous studies have focused mainly on the general characteristics and clinical significance of SEL, with little in-depth research on the specific morphological changes in epidural fat and its direct relationship with RNRs. Our findings indicated that an increase in epidural fat was significantly associated with the presence of RNRs, supporting the hypothesis that increased epidural fat can lead to the occurrence of RNRs, thus providing a new perspective on the early and accurate identification of SEL. This is consistent with the findings of Ishikawa et al. [17], who proposed a grading system for epidural fat but did not directly link it to RNRs. Our study established this connection in a statistically significant manner for the first time, thereby enhancing the understanding of the pathophysiology of SEL.

In Group B, there were three patients with epidural fat graded as I. Further analysis of these patients revealed that Patient 1 had bony spinal canal stenosis, possibly due to congenital developmental abnormalities, which may have been the reason for the occurrence of RNRs. Patient 2 was a diabetic patient with peripheral neuropathy, and whether diabetes is related to the formation of RNRs remains to be studied. Patient 3 only showed RNRs on MRI, and no special circumstances were found in the existing medical history, suggesting that there may have been hidden causes that need further investigation.

Further analysis of the severity of the RNRs in Group B revealed no difference in that the morphologies and relative lengths of the RNRs vary across fat grades; although circular RNRs accounted for more than 90% of the different fat grades, which is the main morphology of the RNRs and is consistent with the literature [18]. Generally, as fat deposition increases and compression time increases, the impact on the cauda equina becomes greater, leading to more severe redundancy; however, the current experimental results did not show differences, possibly because this study did not consider the time factor. In addition, the age range of patients in this group was quite large, making it difficult to determine the time factor in the experimental design. Additionally, the deposition of epidural fat tissue is limited by the bony spinal canal, dural sac, and yellow ligament, so the proportion of patients with a grade of 2 was significantly greater than that with grades of 1 and 3, often manifesting as localized deposition between adjacent pedicles. Although localized deposition compresses the nerve roots, the soft nature of fat tissue limits the compression force, resulting in RNRs that often present as mild cases, specifically relatively short circular RNRs.

Our study has several limitations. First, the sample size was relatively small due to the low incidence of SEL, especially in severe cases, leading to a smaller sample size in Groups B and C, which may affect the generalizability of our findings. Furthermore, this study was conducted at a single center, which may introduce selection bias. Future research should include a larger, multicenter cohort to validate our findings. Second, the retrospective nature of this study means that we cannot control for all potential confounding variables, such as the duration of epidural fat accumulation and its impact on the RNRs. Longitudinal studies are needed to better understand the temporal relationship between increased epidural fat and the development of RNRs.

In summary, SEL is a rare and often overlooked condition, and MRI is the best diagnostic tool. Our findings indicated that increased epidural fat is an important factor in the development of RNRs, suggesting that early MRI screening for epidural fat deposition is crucial to preventing SEL from progressing to more severe neurological symptoms. By incorporating the assessment of epidural fat into routine MRI evaluations, clinicians can more accurately diagnose SEL and implement early interventions, such as weight management and corticosteroid use reduction, to mitigate the risk of RNRs. Additionally, our study provides an imaging-based standard that can be used to stratify patients based on their risk of developing RNRs, thereby informing treatment decisions and potentially reducing the need for invasive surgical interventions.

## Data Availability

The data of this study are not publicly available, but may be requested from the corresponding author upon reasonable request.
